# A Study of Physical Activity Habits of Young Adults

**DOI:** 10.4103/0970-0218.69292

**Published:** 2010-07

**Authors:** Amitav Banerjee, Swati Khatri

**Affiliations:** Department of Community Medicine, DY Patil Medical College, Pune – 411 018, Maharashtra, India. E-mail: amitavb@gmail.com

Sir,

After a decade into the 21^st^ century, we are facing a rising trend of non-communicable diseases associated with sedentary lifestyle. Studies have shown sedentary lifestyles to be associated with an increased risk of cardiovascular diseases (CVD), and all-cause mortality.(1) Some estimates from developed countries indicate that only 15% of the population older than 18 years of age get regular vigorous activity (three times a week for at least 20 min), and 60% report no regular leisure time activity at all, with 25% not active at all.(1) There is also evidence that habits developed in younger ages are likely to track through to later life.(2)

There are indications that we may be fast catching up with the sedentary lifestyles of developed countries.(3)

A cross-sectional study on a random sample of medical students was carried out to find the frequency of various forms of physical activity. The students hailed from middle to upper middle class households and represented the growing affluence of urban Indian society.

A stratified random sample of 21 students of both genders from each term was selected using a table of random numbers. A total study sample of 189 study subjects was selected in this manner. Out of these, 170 agreed to participate in the study giving a response rate of 89.95%.

An instrument (the Aerobics Centre Longitudinal Study Physical Activity Questionnaire) validated in studies in developed countries was used.(4) Questions in the instrument were suitably modified after pretesting.

Out of the 170 respondents, 87 (51.2%) were females and 83 (48.8%) were males. Only 39.4% of the respondents indulged in some vigorous physical activity. There was no association with gender. People with a higher body mass index exercised more frequently.

Table 1 shows the various types of physical activities being indulged in by the study participants. Stair climbing was the most frequent physical activity among the participants. This was a result of lack of choice rather than a voluntary act as the medical college is functioning in a multistorey building. For attending classrooms and visit to the wards, the students have to climb several flights of stairs each day as they are not permitted to use the lifts. Walking was the next frequent physical activity. However, only 12.9% of the people who walked indulged in brisk walking. Only a minority (15–20%) practiced aerobic exercises such as dancing, jogging, or moderate-to-vigorous sports. Weight training was resorted to by 17% of the respondents. An excellent exercise such as swimming was practiced by only 3.53% of the medical students. A common mode of transportation among college students in the previous generation, i.e., bicycling was resorted to by only 9.4% of the study subjects. Majority, 60.6%, did not participate in exercise sessions vigorous enough to work up a sweat at least three times a week. Only 67 or 39.4% of the subjects took part in adequate physical exertion at least three times a week. There was no statistically significant difference in gender and frequency of the physical activity. Paradoxically, in this cross-sectional study it was found that those who were indulging in physical activity more often had a higher mean BMI compared to those indulging in such activity less than three times a week. This brings out one of the limitations of a cross-sectional study, i.e., a temporal association cannot be established. We do not know whether physical activity caused higher BMI or the study subjects with a tendency of obesity and higher BMI started indulging in more physical activity so as to lose weight.

**Table 1:** Different types of physical activity among the study respondents

Type of physical activity	Participations	Percentage
Walking*	82*	48.2*
Stair climbing	147	86.5
Jogging	26	15.29
Treadmill	24	14.1
Bicycling	16	9.4
Aerobic dancing	35	20.6
Moderate sports (volleyball, doubles tennis, etc.)	26	15.29
Vigorous sports (Racquet games, singles’ tennis, etc.)	10	5.88
Other vigorous sports (basketball, football, etc.)	32	18.8
Others (yoga, table tennis)	10	5.88
Weight training	29	17.05
Household activities	50	29.41
Lawn mowing	13	7.65
Swimming	6	3.53

* Only 12.9% who walked indulged in brisk walking

More than 20% of the sample was overweight/obese. A total of 9.4% of the study subjects were underweight. More female subjects (13.8%) were underweight compared to male subjects (4.8%).

To conclude, it was found that the majority of the medical students were not indulging in adequate physical activity. There is a tendency toward sedentary lifestyle among the young people. About one-fifth of the subjects were overweight/obese indicating a shift toward obesity. India will need to implement integrated preventive strategies to address physical inactivity induced by rapid motorization and automation of work-related activities. A comprehensive, intersectoral national plan of action on physical activity promotion for the people is necessary as part of an integrated approach to preventing and controlling non-communicable diseases.

All parts of the body which have a function, if used in moderation and exercised in labors in which each is accustomed, become thereby healthy, well developed and age more slowly, but if unused and left idle, they become liable to disease and defective in growth and age quickly – Hippocrates.

## References

[1] Wong ND, Bassin SL, Wong ND (2000). Physical Activity. Preventive Cardiology.

[2] Kjonniksen L, Torsheim T, Wold B (2008). Tracking of leisure-time physical activity during adolescence and young adulthood: A 10-year longitudinal study. Int J Behav Nutr Phys Act.

[3] Banerjee A (2007). Coronary heart disease: Awareness of risk factors and lifestyle among school-going adolescents. Indian J Med Sci.

[4] Kohl HW, Blair SN, Paffenbarger RS, Macera A, Kronenfeld J (1988). A mail survey of physical activity habits as related to measured physical fitness. Am J Epidemiol.

